# Sick day management in people with chronic kidney disease: a scoping review

**DOI:** 10.1007/s40620-022-01497-5

**Published:** 2022-11-03

**Authors:** Henna Duong, Wubshet Tesfaye, Connie Van, Kamal Sud, Mimi Truong, Ines Krass, Ronald L Castelino

**Affiliations:** 1 School of Pharmacy, Faculty of Medicine and Health, The University of Sydney, Sydney, Australia; 2 Sydney Medical School, Faculty of Medicine and Health, The University of Sydney, Sydney, Australia; 3 Department of Renal Medicine, Nepean Hospital, Nepean and Blue Mountains Local Health District, Katoomba, Australia; 4 Pharmacy Department, Blacktown Hospital, WSLHD, Blacktown, Australia; 5 Sydney Pharmacy School, The University of Sydney, A15, Science Rd , Camperdown, NSW 2006 Australia

**Keywords:** Chronic kidney disease, Medication management, Acute kidney injury, Sick day protocol, Scoping review

## Abstract

**Background:**

Use of certain medications during an acute illness may put patients at an increased risk of acute kidney injury (AKI). Patients with chronic kidney disease (CKD) are at higher risk of developing superimposed AKI. The aim of this scoping review is to collate and characterise existing evidence on sick day management considerations and practices during acute illness in people with CKD.

**Methods:**

We searched Embase, CINAHL, MEDLINE, International Pharmaceutical Abstract, Scopus, Google Scholar and grey literature sources. We followed the methodological framework for scoping reviews, while information was extracted in accordance with the Preferred Reporting Items for Systematic Reviews and Meta-Analyses extension for scoping reviews. Findings are presented thematically.

**Results:**

Ten studies and seven guidelines met the inclusion criteria. Studies were targeted at patients, general practitioners, pharmacists, and nurses. The major themes identified included development and feasibility testing of a sick day management protocol, current practice of temporary medication discontinuation, and outcomes. Most guidelines provided recommendations for sick day management largely based on expert consensus. A digital intervention was deemed highly acceptable and easy to use, whereas patient handouts were more effective when provided along with dialogue with a health professional. While there is little evidence on the impact of sick day protocols on outcomes, a single randomised trial reported no significant association between sick day protocols and change in kidney function, AKI incidents or risk of hospitalisation.

**Conclusion:**

The nascent literature on sick day management in patients with CKD revealed the limited available evidence to provide guidance on implementation and on outcomes. Future research needs to clarify sick day recommendations and assess their impact on clinical outcomes including prevention of superimposed AKI or hospitalisations, as well as to address barriers to implementation.

**Supplementary Information:**

The online version contains supplementary material available at 10.1007/s40620-022-01497-5.

## Introduction

Chronic kidney disease (CKD) is defined based on abnormalities of kidney structure or function lasting for at least three months, with implications for health [1, 2]. CKD is a growing public health problem affecting 8–16% of the adult population worldwide [3]. In Australia, over 1.7 million adults have some biomedical signs of CKD, with the vast majority unaware of their condition. CKD is a cause for significant hospital (re)admission and mortality rates [4]; it was implicated in 16% of overall hospitalisations and 11% of all deaths in the year 2018 alone, costing the Australian economy an estimated $5 billion annually for prevention and management of the disease [4].

Acute kidney injury (AKI), especially in people with pre-existing CKD, is associated with kidney failure as well as increased rates of hospitalisation and mortality [5, 6]. In people with CKD, there are various medical factors that contribute to increased risk of AKI, including reduced homeostatic reserves and the presence of multiple morbidities and associated treatments [7–9]. Importantly, in the absence of appropriate medication or dosage adjustments, the altered pharmacokinetics and pharmacodynamics that occur in CKD predispose patients to an increased risk of adverse drug events [10]. Due to their nephrotoxic nature or potential for causing AKI, certain drug classes are often targeted in interventions to reduce the risk of harm in patients with CKD in the event of acute illness. These include **s**ulfonylureas; angiotensin converting enzyme inhibitors; diuretics; metformin; angiotensin receptor blockers; non-steroidal anti-inflammatory drugs; and sodium glucose co-transporter 2 inhibitors, and are commonly referred to as SADMANS drugs [11].

Temporary discontinuation of high risk medications by patients during acute illness has been proposed as a strategy to reduce the occurrence of AKI in primary care setting. This self-management initiative was first proposed by the National Health Services (NHS) Highland in Scotland in the form of a Medicine Sick Day Rules card and was later adopted by the NHS England to support health care providers communicate the risk of AKI to patients and carers [7]. The goal was to educate and enable patients to identify and temporarily stop these medications themselves to improve safety.

Although there is strong professional consensus and guideline recommendations that promote sick day guidance to temporarily cease certain medications, little is known on the evidence base to support its implementation or clinical relevance [7]. It is apparent that current literature is broad and recent, with limited emerging primary studies conducted on sick day management protocol and its implementation and overall implications on patient care and safety. Therefore, this scoping review aims to characterise and collate evidence from existing literature and guideline recommendations on medication management considerations during acute illness in the context of CKD. The findings of the review will form an evidence base for future research and practical considerations.

## Methods

### Study design

A scoping review was performed to capture the nascent evidence surrounding sick day management in people with CKD. We followed the methodological framework initially proposed by Arksey and O’Malley [12] which was later defined into a five-step process by Levac et al. [13], including: (i) identifying research questions, (ii) identifying relevant studies, (iii) selecting studies, (iv) charting and extracting data, and (v) summarising and reporting results.

### Identifying review questions

The scoping review was guided by the following research questions: (1) What are the characteristics of the published evidence surrounding sick day management of CKD (i.e., the practice of temporary discontinuation of medications during acute illness in people with CKD)? (2) Were interventions focused on sick day management in CKD implemented successfully at the patient or health professional level? and (3) Is there any association between sick day targeted medication discontinuation and clinical outcomes?

For the purposes of this review, “sick day management” was defined as any written or verbal information provided to CKD patients to temporarily cease medication(s) in the event of an acute illness. Any intervention aimed at implementing sick day management advice in a cohort of CKD patients was termed as a “sick day intervention”. These definitions were guided by a position statement from the ‘Think Kidneys Board”, which has been a leading organisation involved in the introduction of the sick day guidance in the United Kingdom [7].

To answer the research questions, a Population, Concept, and Context (PCC) framework proposed by the Johanna Briggs Institute (JBI) Manual for Evidence Synthesis [14] was created, as outlined in Table 1.

**Table 1:** Concepts presented using the population concept, and context framework

Criteria	Element(s)	Description
P- Population	Patients	Individuals at risk of developing AKI on the background of CKD
	Healthcare workers	Healthcare providers such as doctors, nurses, and pharmacists who are involved in CKD care at some stage along the healthcare continuum
C- Concept	Development and usability of sick day rules	Interventions targeted at facilitating the provision of sick day management i.e., the temporary discontinuation of certain medications, commonly referred to as SADMANS medications, that are implicated in AKI and thus need to be withheld upon acute illness to prevent disease deterioration
	Current Practice	Current knowledge, awareness, understanding and attitudes of patients and relevant health professionals towards advice to withhold medications during acute illness
	Current Guidance	Current guidance provided to healthcare professionals on the management of medications during acute illness
	Clinical outcomes	Outcomes that resulted from the use of SADMANS medications or from temporarily withholding/discontinuing them
C- Context	Primary care	Services or interventions developed or implemented in primary care settings
	Any geographical context	Studies from any geographical context that dealt with sick day rules or temporary discontinuation of medications in the context of AKI on top of CKD is considered

Abbreviations: *AKI* acute kidney injury, *CKD* chronic kidney disease, *SADMANS* Sulfonylureas, Angiotensin converting enzyme inhibitors, Diuretics, Metformin, Angiotensin receptor blockers, Non-steroidal anti-inflammatory, Sodium-glucose co-transporter 2 inhibitors

### Identifying studies

Embase, CINAHL, Medline, International Pharmaceutical Abstract (IPA) and Scopus databases were searched in early March 2022, by the primary author (HD), to identify peer-reviewed works published in English. No publication date constraints were applied, and all databases were searched from inception. The applied search strategy was drafted by HD with guidance from an experienced librarian and refined through discussion with the other research investigators (WT, RC, and CV). Subject headings and truncated keywords were guided by the conceptual framework of this study (Table 1). The search was conducted by combining the following major concepts via appropriate Boolean operators: “sick day intervention,” “temporarily discontinuing medications,” “chronic kidney disease,” “acute kidney injury,” and “diabetic kidney disease.” The final search strategy for the different databases is provided in Appendix 1. Citation chaining of included studies was manually conducted to identify any additional publications of relevance which could have been missed during the main database search.

To capture studies and guidelines not indexed in the targeted databases, a grey literature search was performed using Google Scholar. Grey literature sources were also retrieved from consulting organisations focused on CKD, such as the Kidney Disease: Improving Global Outcomes (KDIGO), Kidney Health Australia, and National Kidney Foundation and Diabetes Canada.

### Study selection

Studies were included if they were peer-reviewed journal papers published in English and explored sick day management aimed at preventing AKI in the context of pre-existing CKD. All quantitative, qualitative or mix-methods studies except case reports were considered for inclusion so as to capture the broad evidence surrounding sick day management in people with CKD. Studies were excluded if the described intervention did not target CKD patients or were interventions applied in the instance of known or suspected AKI.

Guidelines were included if they provided a recommendation to temporarily discontinue medications to prevent AKI in patients with kidney disease. Guidelines providing a recommendation to temporarily discontinue medications to prevent other outcomes such as lactic acidosis were excluded as this was not within the scope of the review.

All studies identified through database searches were exported to EndNote™ 20 and subsequently transferred to Covidence [15]. After removal of duplicates, the remaining studies were screened using title, abstract and full text. We considered both quantitative and qualitative studies that explored sick day management or discussed temporary discontinuation of medications in acute illness as a key concept. Perspective/opinion pieces, letter to editor, conference abstracts, clinical trial registrations and case studies were excluded. Guidelines recommending sick day management or temporary discontinuation of medications to prevent AKI in patients with CKD were eligible for inclusion. Documents were independently screened against the eligibility criteria by two authors (HD and WT) and disagreements were resolved through in-depth discussion among research investigators (HD, WT and RC).

### Data charting and extraction

The primary investigator (HD) developed an initial data charting table which was later discussed and approved by all authors. The following items were extracted from included studies: author, year and country of publication, study, participant characteristics, concepts explored, perspective explored, aim of study, type of intervention, details of sick day guidance, summary of findings, and major limitations reported. Data from guidelines were presented using a separate extraction table developed specifically for the guidelines. This was done to ensure that key concepts relevant only to guidelines were clearly reported. Data extracted from guidelines included: endorsing organisation or body, year and country of publication, recommendations, evidence and/or rationale and strength of recommendation.

### Data summary and presentation of results

Narrative descriptions of the extracted data were analysed using thematic content analysis, and results were organised under the following four themes: Development and usability of sick day rules, current practice by patients or health professionals, current guideline recommendations and clinical outcomes. The review is reported in accordance with the Preferred Reporting Items for Systematic Reviews and Meta-Analyses extension for scoping reviews (PRISMA-ScR) (Appendix 2) [16].

## Results

### Description of studies

**Fig. 1:**
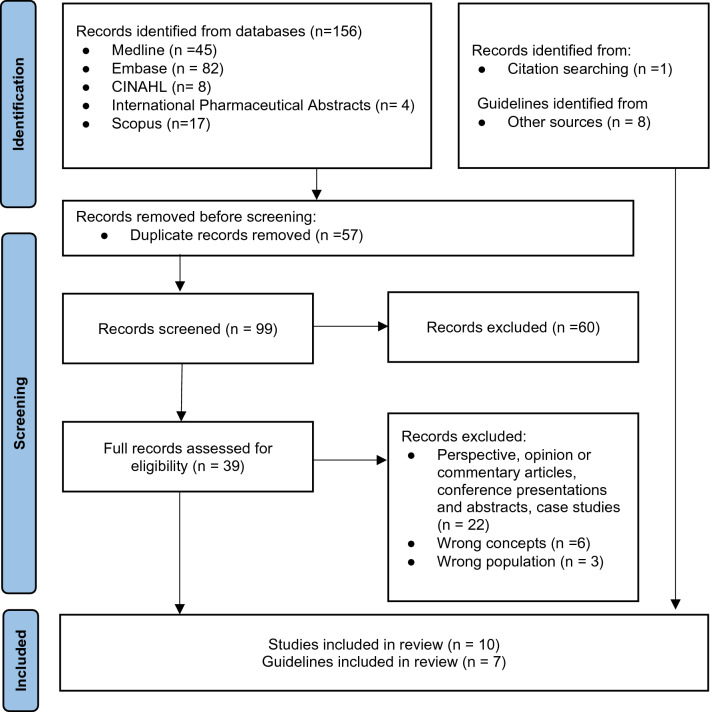
PRISMA 2020 flow diagram for new systematic reviews which included searches of databases, registers and other sources. *Consider, if feasible to do so, reporting the number of records identified from each database or register searched (rather than the total number across all databases/registers). **If automation tools were used, indicate how many records were excluded by a human and how many were excluded by automation tools. From: Page et al. [41]. For more information, visit: http://www.prisma-statement.org/

A total of 156 studies were retrieved from the electronic database searches. After removing duplicates, a total of 99 studies were considered for initial screening. Title and abstract screening against the inclusion criteria yielded 39 studies. Following full text screening, eight studies met the inclusion criteria and were included in this review. Supplementary searches of citations provided two additional primary studies, providing a total of 10 works for final inclusion in the review. Search of grey literature sources resulted in the inclusion of seven guidelines. Figure 1 presents the PRIMSA flow diagram for study inclusion process.

### Characteristics of included studies

Of the 10 included studies, [17–25] eight were original articles [17–22, 25, 26], one was a letter to the editor but contained sufficient original data [24] and one was a systematic review [23]. Table 2 shows the characteristics of the included studies, while Appendix 3 provides additional study attributes such as major findings and study limitations. The most common primary study design was a mixed-method design (40%) followed by quantitative design (30%) and qualitative design (20%). The patient’s perspective was the most reported in the included studies (70%), but studies also considered health care professional perspectives (pharmacists, general practitioners, and nurses). Two studies (20%) included both patient and healthcare professional perspectives. Five studies (50%) explored the development, usability and/or implementation of sick day interventions and three studies (30%) focused on current practice of providing sick day management advice. A randomised controlled trial, [25] an observational primary study [22] and a systematic review [23] explored outcomes. Studies were carried out in 4 countries.

**Table 2:** Study characteristics summary (excluding guidelines)

Characteristics	Number of studies (%)	Author
Publication year		
2022	1 (10)	Fink et al. [25]
2020	4 (40)	Vicary et al. [17], Vicary et al. [26], Vicary et al. [18], and Bowman et al. [20]
2019	2 (20)	Faber et al. [22] and Doerfler et al. [24]
2017	2 (20)	Martindale et al. [21] and Whiting et al. [23]
2016	1 (10)	Morris et al. [19]
Study by country/region		
United States	3 (30)	Bowman et al. [20], Doerfler et al. [24] and Fink et al. [25]
United Kingdom	3 (30)	Morris et al. [19], Martindale et al. [21] and Whiting et al. [23]
Netherlands	1 (10)	Faber et al. [22]
New Zealand	3 (30)	Vicary et al. [17], Vicary et al. [26] and Vicary et al. [18]
Types of articles		
Original articles	8 (80)	Vicary et al. [17], Vicary et al. [18], Morris et al. [19], Bowman et al. [20], Martindale et al. [21], Faber et al. [22], and Vicary et al. [26]
Letter to the editor^*^	1 (10)	Doerfler et al. [24]
Systematic review	1 (10)	Whiting et al. [23]
Study designs		
Quantitative	2 (20)	Vicary et al. [17] and Faber et al. [22]
Qualitative	2 (20)	Morris et al. [19] and Martindale et al. [21]
Mixed methods research	4 (40)	Vicary et al. [26], Vicary et al. [18], Bowman et al. [20] and Doerfler et al.[24]
Systematic review	1 (10)	Whiting et al. [23]
Randomised controlled trial	1 (10)	Fink et al. [25]
Perspectives explored**		
Patients	6 (67)	Vicary et al. [17], Vicary et al. [26], Morris et al. [19], Bowman et al. [20], Martindale et al. [21] and Doerfler et al. [24]
Pharmacists	3 (33)	Vicary et al. [18], Morris et al. [19] and Martindale et al. [21]
General practitioner	4 (44)	Vicary et al. [18], Morris et al. [19], Martindale et al. [21] and Faber et al. [22]
Practice nurses	2 (22)	Morris et al. [19] and Martindale et al. [21]
Key concepts***		
Development, usability, or implementation of “sick day” guidance	5 (50)	Vicary et al. [26], Morris et al. [19], Bowman et al. [20], Martindale et al. [21], Fink et al. [25] and Doerfler et al. [24]
Current practice	3 (30)	Vicary et al. [17], Vicary et al. [18] and Faber et al. [22]
Outcomes	2 (20)	Whiting et al. [23], Faber et al. [22] and Fink et al. [25]

^*^Included primary study reported in a Letter to the Editor paper

^**^Some studies explored multiple perspectives

^***^Some studies explored multiple concepts

### Development/usability testing and implementation of sick day management

Six studies [19–21, 24–26] explored the theme of development and implementation of interventions to provide sick day management (Table 3).

**Table 3:** Summary of findings from included studies

Key concept	Evidence	Exemplary findings
Development, usability testing and implementation of sick day guidance		
Limited number of interventions explored	Vicary et al. [26]	• Pharmacists were effective educators and well placed in the community to provide sick day advice during acute illness • 58% (*n* = 54) participants interviewed on follow up recall receiving the intervention and 55% (n = 42) had retained the guidance sheet provided
	Morris et al. [19]	• Patient handouts alone were seen as insufficient and unlikely to improve outcomes. The implementation of sick day interventions likely requires more resources in the form of remuneration and overall health infrastructure
	Bowman et al. [20]	• Majority of participants found a digital tool easy to use, helpful and would recommend it to others • Participants identified story examples and guiding audio as important components to aid in understanding of sick day management • Digital education tools may be effective in educating CKD patients with low health literacy and/or older age
	Doerfler et al. [24]	• The use of index cards containing information on sick day management did not appear effective and raised concerns regarding consequences of incorrect activation leading to harm
	Fink et al. [25]	• The use of a sick day protocol coupled with an interactive voice response system to enable event reporting was associated with high engagement with participants, although this did not translate into better clinical outcomes
	Martindale et al. [21]	• As a stand-alone intervention, sick day guidance cards may have little benefit • Guidance cards should be used to supplement patient education, but HCPs reported this did not regularly occur
Barriers to implementation of sick day management protocol	Vicary et al. [17]	• Whilst half of patients who recalled receiving sick day management advice indicated they would discontinue medications when they were sick, less than 20% of patients who recalled the advice would correctly discontinue medications if they had “excessive vomiting and/or diarrhoea.”
	Vicary et al. [26]	• Around half of participants would discontinue medications during acute illness but may not cease the correct medications • More than half of participants were comfortable that they knew when to restart their medications but only 16% would restart medications after being symptom-free for 48 h per recommendations
	Vicary et al. [18]	• Pharmacists and GPs highlighted that whilst instructions seemed straightforward, they require a high level of knowledge by patients
	Doerfler et al. [24]	• Whilst most participants were able to correctly identify an index scenario where the “sick day” guidance should be applied, many patients activated the “sick day” guidance for scenarios where it was inappropriate to do so • Most participants (95%) made errors when selecting appropriate medications to discontinue during acute illness
	Martindale et al. [21]	• HCPs had concerns about the depth of understanding of symptoms and medications required by patients to proactively discontinue medications during acute illness • HCPs were wary of the subjective nature of guidance, highlighting that patients had different perceptions on what is “severe” illness • Implementing sick day management may be difficult in patients who have cognitive impairments, reduced literacy in English, visual impairments, or elderly housebound patients
	Morris et al. [19]	• Patients were uncertain if they would be able to distinguish between symptoms of various conditions which may affect their ability to assess the appropriateness of sick day management
Current practice		
Rate of sick day management advice	Faber et al. [22]	• 91% of GPs did not offer high risk patients advice to discontinue or adjust medication dosages or referred them to hospital during a dehydration risk encounter
	Vicary et al. [18]	• Only 16% of pharmacists and 11% of physicians (GPs) reported that they always provided sick day management advice to patients prescribed an ACEI/ARB/NSAID/diuretic
	Vicary et al. [17]	• Around 14% of participants taking an ACEI, ARB, diuretic, NSAID and/or metformin indicated that they had been advised by an HCP to stop taking medicines during acute illness
Coordination of care and defined roles	Vicary et al. [18]	• There are varied and unclear expectations of the level of patient education that should be provided by pharmacists – over half of GPs expected “sick day” guidance to be provided by pharmacists but pharmacists do not report routinely providing this advice • Barriers identified by pharmacists included time constraints, unclear renumeration and lack of existing GP and pharmacist collaboration • Pharmacists required clear support from GPs – they will only provide advice if GPs view it as best practice • GPs preferred to be contacted to discuss their patients’ condition or at least informed when discontinuation advice was provided
	Morris et al. [19]	• Both patients and HCPs highlighted the need for a consistent message about sick day rules • Patients and HCPs questioned which provider should be providing the sick day management advice • Legal and professional boundaries limited the willingness of nurses and pharmacists to implement sick day rules • A key barrier to pharmacists providing targeted “sick day” guidance is that they had limited access to diagnostic information including patients’ renal function
Outcomes		
Limited evidence on outcomes associated with medication discontinuation	Whiting et al. [23]	• The systematic review identified no published literature reporting on the impact of temporarily discontinuing medications during acute illness
	Faber et al. [22]	• In 3.1% (n = 25) of episodes of acute illness, a complication occurred in the subsequent 3 months after contacting their GP, most commonly AKI. In 88% of these cases, no discontinuation advice was provided • In three episodes the patients had been advised to discontinue their high-risk medication, but despite this advice AKI (n = 2) or hypotension (n = 1) occurred
	Fink et al. [25]	• When adjusted for baseline eGFR, there is no statistically significant difference in mean change of eGFR in a 6-month period between patients who received a sick day protocol handout and weekly survey calls from an interactive voice response system (intervention group) and patients who received usual care • There were no statistically significant differences in number of hospitalisations, emergency department or urgent care visits • Only half of sick day events (n = 33) reported through the interactive voice response system were true sick day events • In the instance of a true sick day event, only half of the participants in the intervention group correctly discontinued their medication(s) • There were high rates of engagement reported and high ease of use, ease of comprehension and desire to continue using the program • There was notable error in the use of the digital tool both in the identification of a true sick day event and correctly applying the sick day protocol

Abbreviations: *ACEI* angiotensin converting enzyme inhibitor, *ARB* angiotensin receptor blocker, *AKI* acute kidney injury, *CKD* chronic kidney disease, *GP* general practitioner, *HCP* healthcare professional, *NSAID* non-steroidal anti-inflammatory drug

#### Limited number of sick day interventions

The most common method for providing sick day intervention information was a patient handout (pamphlet, information sheets, cards) [19, 21, 24–26]. Three studies combined patient handouts with advice from a health care professional [19, 21, 26]. One study trialled the use of a digital tool to educate patients with CKD on sick day management [20]. One of the studies used a card (a fold-over business card) containing a list of harmful medications coupled with a designated telephone number (wireless or landline) and an interactive voice response system for reporting sick day events as part of the intervention [25].

Sick day interventions that were trialled reported varying degrees of usability and acceptability [19–21, 24, 26]. For instance, one study that tested the usability of a digital tablet-based program in older adults with CKD found that sick day management education utilising clinical vignettes reported high acceptability and ease of use [20]. Fink et al. also reported high levels of acceptance and engagement of participants with an interactive voice response system that followed the use of a sick day intervention card [25]. In contrast, another study reported a considerable error rate with the usability testing of a sick day management card to correctly identify medications to be avoided to prevent AKI [24]. One study reported that up to a third of participants (*n* = 28) provided positive feedback and half of them (*n* = 51) had retained the sick day guidance sheet developed as part of the intervention [18].

#### Barriers to implementation of sick day guidance

Interestingly, most of the studies that focused on development or testing of interventions agreed that the implementation of a patient handout, especially as a stand-alone intervention, was likely to offer little benefit [19, 21, 24]. This was reflected by a patient who emphasised the passive nature of patient handouts:

“I don't think that it should be just put on a counter… I don't think, number one, they’ll read it, number two, they’ll digest what’s on it, or number three, they’ll apply it to themselves…” [21]

This is in line with five studies identifying low patient health literacy as a barrier to sick day management [17, 19, 21, 24, 26]. Studies reported that patients regularly made errors when identifying appropriate scenarios to temporarily discontinue their medications and made errors in selecting appropriate medications to discontinue [17, 24, 26]. Additional barriers to the implementation of sick day management include communication and cognitive functioning [17, 19, 21, 24, 26]. People with communication barriers are more likely to have an additional challenge understanding the messaging from health professionals. This was outlined by one nurse practitioner:

“we have quite a lot of different ethnicities here…they’ve got limited English I think they’re not quite sure and it takes quite a while explaining …about what medicines to stop, when to stop it, when to restart it…” [21]

Three studies reported that while sick day guidance can be successfully implemented in primary care settings, the additional workload associated with implementing such protocols and integrating counselling into routine practice was a barrier [18, 19, 21]. Some pharmacists and practice nurses indicated that sick day management was often not provided simply due to a lack of time and growing workload [18, 19, 21]. A practice nurse expressed:

“You’ve got to look at everything else that’s going on…you get patients obviously with multiple medical problems and you must try and remember to include everything in your consultation. It’s sometimes quite hard” [19].

### Current practices on the provision of sick day management

Three studies [17, 18, 22] explored the current rates of sick day management being provided to patients who are at greater risk of AKI due to either risk factors such as CKD and/or medication usage (Table 3).

#### Poor uptake of sick day guidance

One study [17] outlining patient experiences showed that less than 15% of patients taking an at-risk medication (ACEI, ARB, diuretic, NSAID and/or metformin) indicated that they were provided advice on actions to be taken during periods of acute illness.

Two studies mapped the healthcare professional’s perspectives [18, 22]. Some general practitioners (GPs) did not recommend that patients discontinue at-risk medications during an acute illness, nor was education on sick day management offered regularly when medications were prescribed [18, 22]. Faber et al. [22] reported that in over 91% of cases in which patients contacted their doctor with an acute illness, no sick day management advice was given.

#### Lack of evidence and poor information access

Two studies suggested that the poor provision of sick day management advice was partly due to the lack of published evidence surrounding the best practice around temporarily discontinuing medications [18, 21]. There is uncertainty about exactly when to stop and restart a medicine and other dosage considerations. This is explained by one GP as follows:

“We don’t have enough data or…best practice… if you stop the metformin or whatever medication how long do you stop it for…? Then after a week are you going to restart them again on the ten milligrams or are you going to start them on the 1.5, the 2.5…?” [21]

Two studies reported that pharmacists believe, despite the lack of access to relevant clinical information (especially diagnostic findings), that they can effectively provide information relevant to AKI prevention while dispensing medication, particularly in the presence of a more integrated primary care model [18, 21]. One pharmacist articulated:

“I think as pharmacists we could deliver it in a positive way because we’d have the time to sit with the patient, do a medicines review, or even without a medicines review the fact is that when we’re dispensing any medication you’ve got time to really engage with them, probably more so than the GP would… But identifying the patients in the first place would be a big stumbling block for us.” [19]

Vicary et al. [18] reported that over 72% of pharmacists (*n* = 23) were willing to participate in sick day management.

#### Importance of coordination of care and defined roles

Two studies reported on the importance of collaboration and coordination of care between GPs and other health services [18, 19]. It was considered essential that all health professionals provide the same advice, *“…everyone pulling together and giving the same message, including the receptionist”* [19]. Furthermore, pharmacists highlighted the need for collaboration and that *“advising on temporary discontinuation of medications … should be collaborative with the GP so they are aware…”* [18] GPs also valued this communication, reporting that they “…*would want to be involved in the discussion”* [18].

In addition to coordination of care, any intervention or practice improvement should have clarity around professional boundaries and roles to avoid any uncertainties patients may have regarding where to go for guidance. This was reported as a necessity by both patients and health care professionals in two studies [18, 19]. One patient’s concern was succinctly highlighted as follows, *“who should it come from, is it the GP or would it be the nurse or the pharmacist?”* [19] Pharmacists expressed that they would not provide this advice *“unless prescribers regarded [sick day management] as best practice….”* [18] A practice nurse echoed the need for coordination and clarity of roles stating that *“I would consult the doctor … no court in the land would support me. It would be very wrong ….”* [19] Some patients also indicated limited confidence in pharmacists’ advice due to lack of access to important patient information:

*“GP or nurse…I suppose a GP or the nurse at the GP centre would know more about my history than the pharmacist would, so I would be more likely to take their word for it.”* [19]

### Outcomes

We found three studies (two primary studies and a systematic review) [22, 23] that assessed the association between medication (dis)continuation and outcomes/complications. One randomised trial (*n* = 159 and *n* = 156 in intervention and usual care, respectively) examined the link between a sick day protocol and change in kidney function in high-risk patients with CKD. The study revealed no difference in adjusted mean change in estimated glomerular filtration rate (eGFR) between the sick-day protocol (− 0.71; 95% CI − 2.11 to 0.69 mL/min/1.73 m^2^) and usual care (− 0.72; 95% CI − 2.12 to 0.68) groups from baseline to 6-months follow-up [25]. The mean differences in eGFR also showed no significant difference between the groups after adjusting for baseline eGFR (*P* = 0.99). Additionally, there were no significant differences in secondary outcomes, such as rates and frequency of hospitalisations, and emergency department and urgent care visits [25].

An observational study by Faber et al. [22] reported an association between the provision of sick day management and overall disease complications (Table 3). This study reported low rates of advice given by GPs on medication discontinuation or adjustment – 88% of patient episodes had not received prior sick day guidance from a GP. Additionally, this study reported three clinical episodes where patients had been advised to discontinue high-risk medications but nonetheless ended up developing a complication (AKI or hypotension) [22].

One systematic review [23] that included six studies (three observational and three randomised controlled trials) targeting 1663 participants with a mean age range of 65–73 years explored benefits and harms associated with discontinuing medications to prevent AKI. This review only found studies that were conducted in hospital settings. The review highlighted that people with intercurrent illness who continued ACEIs/ARBs were not at significantly increased risk of AKI or contrast-induced nephropathy (relative risk 1.17; 95% confidence interval 0.99–1.38). The evidence from three randomised trials alone also revealed no statistically significant association between continued therapy with ACEIs/ARBs and the risk of AKI (relative risk 1.48; 95% confidence interval 0.84–2.60). However, it is important to note that, per the authors of this systematic review, the evidence from the randomised trials and the observational studies were rated as low and very low quality, respectively [23].

### Summary of findings from guidelines

In addition to the original studies identified above, seven guidelines that described or mentioned sick day guidance in people with CKD were reviewed [27–33]. The detailed characteristics and recommendations of the included guidelines are provided in Appendix 4. Most guidelines that recommended a form of sick day management or temporary discontinuation of medications during acute illness, based their recommendations on expert consensus. Most guidelines did not indicate the strength of their recommendation (i.e., how important the experts believed the implementation of the recommendation to be); however, three guidelines [27, 29, 31] recommended providing most patients with guidance regarding sick days as apparent benefit is likely to outweigh risk. None of the guidelines clearly indicated the evidence base that was considered and only four provided a clear rationale for their recommendations. From these four guidelines [28–31], the consensus appears to be that continuation of certain classes of medications during illness is associated with volume depletion and that this may lead to AKI based on the medication’s pharmacokinetic and/or pharmacodynamic characteristics.

## Discussion

This review presents published evidence on the practice and interventions surrounding sick day management in patients with CKD. While some guidelines recommend temporary cessation of high-risk medications on sick days, original studies highlighted ambiguities and uncertainties among health professionals and patients translating such recommendations into practice. To the authors’ knowledge, this is the first scoping review to summarise practices and challenges in implementing sick day management solely focused on CKD. We found one recent scoping review [34] that characterised the evidence on sick day management guidance for people with diabetes, kidney, or cardiovascular diseases. However, we uniquely applied the PCC framework to identify common themes from quantitative and qualitative studies to understand existing research gaps. Also, we included additional studies [17–19, 22, 23] which were not part of the previous scoping review [34]. Finally, our focus on CKD allowed an in-depth discussion of the implementation of sick day management protocols and the associated challenges in this population.

Despite the limited evidence on temporary medication cessation during acute illness, some guidelines provided sick day management guidance which was developed through expert consensus and based on the following notions: (1) the mechanism of action of certain medications may predispose patients to an increased risk of AKI, (2) medications that are cleared by the kidneys could accumulate during periods of decreased kidney function, and (3) continuing use of certain medications during periods of acute illness may lead to poor outcomes. Whilst these rationales appear logical and sound, guidelines highlighted the lack of clear evidence to support such recommendations and the considerations are based largely on the trade-off between clinical benefit and harm [28, 33].

Guidelines discussed medication discontinuation during acute illness, but the definition of illness varies across guidelines. For instance, the KDIGO 2012 Clinical Practice Guideline defines it as “serious intercurrent illness that increases risk of AKI,” [29] whereas Kidney Health Australia defines it as when “[patients are] ill and are unable to maintain adequate fluid intake due to gastrointestinal upset or dehydration” [32]. The lack of clarity and consistency from current guidelines has likely impeded the widespread adoption of the guidance and may explain the low rates of advice being given by health care professionals [22]. This finding has to be seen in light of the barriers previously identified by primary care health professionals around the use of CKD identification and management guidelines which were reported to be difficult to use, inconsistent and involve frequently changing evidence [35].

Our findings from original studies identified a lack of cohesion, communication, and clarity among various health care providers when approaching sick day management in CKD. Vicary et al. [18] found that GPs expect sick day guidance to be provided by pharmacists although pharmacists are less likely to provide this guidance. This was attributed to lack of time and remuneration, concerns around consumer health literacy and limited GP-pharmacist collaboration [18, 19]. The poor coordination of care was acknowledged as a problem in CKD care overall [36, 37]—patients’ experience is complex and inconsistent, which, at times, results in conflicting advices from various health care providers. Interestingly, GPs reported confidence that pharmacists can deliver sick day guidance and pharmacists reported having the required skills and knowledge to do so. While most patients accepted pharmacist-led education, some believed that there was a need for additional clinical feedback from clinicians prior to implementing sick day protocols in pharmacy context [26]. Primary care initiatives were explored by the NHS England and Scotland through dissemination of sick day rules cards by health professionals, including pharmacists, with the goal to prevent AKI [7, 38]. In Australia, there is a lack of evidence on the involvement of pharmacists in the provision of sick day guidance in people with chronic conditions including CKD. However, growing collaboration between pharmacies and general practices in various primary care initiatives [39] may create an avenue for future studies to trial an integrated service model with the view to improve sick day guidance in CKD. The use of community pharmacies, as the most highly accessible and convenient health resource, particularly provides a unique opportunity to identify and prevent the occurrences of AKI, potentially reducing the risk of preventable hospitalisations and healthcare costs.

While sick day interventions were administered to patients in the form of handouts or via a digital platform, the latter seemed to be more successful in assisting patients to effectively identify high risk medications. Nevertheless, these studies also reported common barriers to implementation of sick day management protocols. At the core of sick day medication management is enabling and empowering patients to self-manage their conditions in the long run. Patients with CKD are expected to recognise the signs of acute illness and assess the severity of their symptoms to determine if they should temporarily discontinue their medications. However, poor patient health literacy was one of the challenges reported by patients and healthcare professionals alike, which affects the implementation of sick day protocols [21]. This echoes the literature exploring self-management in patients with CKD which reports that poor health literacy is a key barrier to the success of self-management interventions [40]. Health literacy impacts patients’ understanding of the importance of sick day management and adherence to guidance, potentially compounding the risk of complications during an acute illness. This may partly explain the findings that patient handouts alone had limited success in improving self-management unless coupled with explanations by health professionals. Future interventions for sick day management should focus on innovative approaches that are applicable to people with different levels of health literacy. Further, the usefulness of utilising health information systems to implement some of these strategies should also be investigated.

Our review identified a clear evidence gap with regard to clinical outcomes associated with temporary discontinuation of medications or sick day protocol intervention during acute illness in people with CKD. Evidence from one randomised trial reported that implementation of a self-management sick day protocol was not associated with improvement of short-term kidney function or hospitalisation rates [25]. However, this study was limited due to the low overall incidence of sick days recorded over the study period, potentially attributed to the relatively small sample size [25]. Other outcome-related evidence comes from a systematic review that examined the link between continued use of ACEIs/ARBs and AKI incidents [23]. Although there were trends indicating a higher probability of AKI with continued ACEI/ARB use on sick days, the studies were underpowered and must be interpreted cautiously. Also, the review, which is based on low or very low-quality studies, reported only evidence from a hospital setting, thereby limiting its generalisability to community settings. Interestingly, one retrospective study found that complications still occurred despite patients receiving medication discontinuation advice [22]; however, this too was a small descriptive study without any inferential statistical analysis. Overall, our understanding of the association between temporarily withholding high-risk medications in people with CKD and clinical outcomes remains very limited, particularly in the primary care setting. There is a need for further evidence, ideally from additional randomised trials or large-scale prospective studies, to understand the effect of implementing sick day protocols in people with CKD.

### Limitations

This scoping review provides a general overview of the current state of literature surrounding the concept of medication management during acute illness in people with CKD, however, the limited evidence in this area highlights the need for more research. Particularly, the effect of medication discontinuation as part of sick day protocols on clinical outcomes remains less understood. Evidence is particularly lacking on the clinical and safety implications of sick day management protocols in community settings. Secondly, the participants’ characteristics were not universally reported throughout the primary studies and hence were not summarised in the scoping review, which may limit our understanding of the detailed sociodemographic characteristics of the patients targeted in the interventions. This was a key gap in our review as the reported benefit of different interventions may be influenced by various participant factors including level of literacy, age, cognitive functioning, and educational background.

## Conclusions

Patients with CKD do not often receive advice to discontinue high-risk medications that may lead to adverse events like AKI during an acute illness. There appears to be limited evidence on the association between temporary (dis)continuation of high-risk medications and outcomes, particularly in primary care settings. Current recommendations by guidelines on sick day rules primarily stem from expert consensus and the translation of such recommendations into practice remains largely unclear. While current strategies on the provision of sick day guidance resulted in conflicting results, the importance of such guidance on outcomes such as prevention of superimposed AKI or hospitalisations remains poorly understood. Giving patient handouts was the most common form of providing advice, although this strategy resulted in limited benefit when implemented as a stand-alone intervention. Collectively, the existing evidence indicates the need for more research to better understand the role of sick day management principles, barriers to implementation and their relevance in determining outcomes.

## Supplementary Information

Below is the link to the electronic supplementary material.

Supplementary file1 (DOCX 55 kb)
