# Supplementary material for: Sick day management in people with chronic kidney disease: a scoping review
Source: J Nephrol. 2022 Nov 3;36(5):1293–306. doi: 10.1007/s40620-022-01497-5 (PMC10333414; doi:10.1007/s40620-022-01497-5)
Supplement: Supplementary file 1 — Supplementary file1 (DOCX 55 kb) [file 40620_2022_1497_MOESM1_ESM.docx]

**APPENDIX 1:** Search Strategy

**Search Strategy for EMBASE**

(exp chronic kidney failure/ OR chronic kidney* OR chronic renal* OR renal insuffici* OR CKD OR diabetic nephropathy OR diabetic kidney disease* OR AKI OR acute kidney* OR acute renal*) AND (sick day guide* OR sick day rule* OR sick day protocol* OR sick day manage* OR sick-day* OR temporar* discontinu* OR temporar* ceas*)

**Search Strategy for MEDLINE**

(exp Renal Insufficiency, Chronic/ OR chronic renal insufficiency* OR chronic renal* OR chronic kidney* OR CKD. OR AKI OR acute kidney* OR acute renal* OR Diabetic Nephropathies/ OR diabetic nephropath* Or diabetic kidney*) AND (sick-day* OR sick day* OR sick day protocol* OR sick day manage* or sick day rule* OR tempoar* ceas* OR temporar* with* OR temporar* discontinu*)

**Search strategy for CINAHL**

(MH "Kidney Failure, Acute+" OR AKI OR "acute kidney" OR "acute renal") AND ("sick day" OR "sick-day" OR temporar* discontin* OR tempoar* ceas*) AND (CKD OR (MH "Kidney Failure, Chronic+") OR (MH "Renal Insufficiency, Chronic+") OR (MH "Renal Insufficiency+"))

**Search strategy for INTERNATIONAL PHARMACEUITCAL ABSTRACTS**

(sick-day* OR sick day* OR sick day protocol* OR sick day management* OR sick day rule* OR sick day guid*. OR temporar* ceas* OR temporar* discontinu* OR temporar* withh*.mp.) AND (chronic renal insufficien* OR chronic renal failure* OR chronic kidney failure* OR chronic kidney diseas* OR CKD OR AKI OR acute renal* OR acute kidney OR diabetic nephropath* OR diabetic kidney*)

**Search strategy for SCOPUS**

TITLE-ABS-KEY ("*AKI" OR "acute kidney*" OR "acute renal*")
AND TITLE-ABS-KEY ( "sick day*" * OR "sick-day*" )
OR TITLE-ABS-KEY ( temporar* AND ( discontin* OR cease* OR withhold* OR withheld ) )
AND TITLE-ABS-KEY ( "CKD" OR "chronic kidney*" OR "chronic renal *" )

**APPENDIX 2:** Preferred Reporting Items for Systematic reviews and Meta-Analyses extension for Scoping Reviews (PRISMA-ScR) Checklist

| **SECTION** | **ITEM** | **PRISMA-ScR CHECKLIST ITEM** | **REPORTED ON PAGE #** |
| --- | --- | --- | --- |
| **TITLE** | | | |
| Title | 1 | Identify the report as a scoping review. | 1 |
| **ABSTRACT** | | | |
| Structured summary | 2 | Provide a structured summary that includes (as applicable): background, objectives, eligibility criteria, sources of evidence, charting methods, results, and conclusions that relate to the review questions and objectives. | 1 |
| **INTRODUCTION** | | | |
| Rationale | 3 | Describe the rationale for the review in the context of what is already known. Explain why the review questions/objectives lend themselves to a scoping review approach. | 2-3 |
| Objectives | 4 | Provide an explicit statement of the questions and objectives being addressed with reference to their key elements (e.g., population or participants, concepts, and context) or other relevant key elements used to conceptualize the review questions and/or objectives. | 3 |
| **METHODS** | | | |
| Protocol and registration | 5 | Indicate whether a review protocol exists; state if and where it can be accessed (e.g., a Web address); and if available, provide registration information, including the registration number. | N/A |
| Eligibility criteria | 6 | Specify characteristics of the sources of evidence used as eligibility criteria (e.g., years considered, language, and publication status), and provide a rationale. | 3 |
| Information sources | 7 | Describe all information sources in the search (e.g., databases with dates of coverage and contact with authors to identify additional sources), as well as the date the most recent search was executed. | 4 |
| Search | 8 | Present the full electronic search strategy for at least 1 database, including any limits used, such that it could be repeated. | Appendix 2 |
| Selection of evidence sources* | 9 | State the process for selecting sources of evidence (i.e., screening and eligibility) included in the scoping review. | 4, Fig 1 |
| Data charting process** | 10 | Describe the methods of charting data from the included sources of evidence (e.g., calibrated forms or forms that have been tested by the team before their use, and whether data charting was done independently or in duplicate) and any processes for obtaining and confirming data from investigators. | 4-5 |
| Data items | 11 | List and define all variables for which data were sought and any assumptions and simplifications made. | 5, Table 1 |
| Critical appraisal of individual sources of evidence | 12 | If done, provide a rationale for conducting a critical appraisal of included sources of evidence; describe the methods used and how this information was used in any data synthesis (if appropriate). | N/A |
| Synthesis of results | 13 | Describe the methods of handling and summarizing the data that were charted. | 5 |
| **RESULTS** | | | |
| Selection of sources of evidence | 14 | Give numbers of sources of evidence screened, assessed for eligibility, and included in the review, with reasons for exclusions at each stage, ideally using a flow diagram. | 6, Fig 1 |
| Characteristics of sources of evidence | 15 | For each source of evidence, present characteristics for which data were charted and provide the citations. | 6, Table 2 |
| Critical appraisal within sources of evidence | 16 | If done, present data on critical appraisal of included sources of evidence (see item 12). | N/A |
| Results of individual sources of evidence | 17 | For each included source of evidence, present the relevant data that were charted that relate to the review questions and objectives. | 6-10, Table 2 &3, Appendix 3 |
| Synthesis of results | 18 | Summarize and/or present the charting results as they relate to the review questions and objectives. | 6-10, Table 3 |
| **DISCUSSION** | | | |
| Summary of evidence | 19 | Summarize the main results (including an overview of concepts, themes, and types of evidence available), link to the review questions and objectives, and consider the relevance to key groups. | 11-13 |
| Limitations | 20 | Discuss the limitations of the scoping review process. | 13 |
| Conclusions | 21 | Provide a general interpretation of the results with respect to the review questions and objectives, as well as potential implications and/or next steps. | 14 |
| **FUNDING** | | | |
| Funding | 22 | Describe sources of funding for the included sources of evidence, as well as sources of funding for the scoping review. Describe the role of the funders of the scoping review. | N/A |

*Evidence source broadly defined to capture heterogenous data sources (e.g., quantitative and/or qualitative research, systematic review guidelines).

* The JBI guidance as well as Arksey and O’Malley and Levac et al refer define data extraction in a scoping review as data charting*.*

**Reference:** Tricco AC, Lillie E, Zarin W, O'Brien KK, Colquhoun H, Levac D, et al. PRISMA Extension for Scoping Reviews (PRISMAScR): Checklist and Explanation. Ann Intern Med. 2018;169:467–473. [doi: 10.7326/M18-0850](http://annals.org/aim/fullarticle/2700389/prisma-extension-scoping-reviews-prisma-scr-checklist-explanation)

**APPENDIX 3:** Characteristics of included studies

| **Author, Year, Country, Reference** | **Design** | **Participant Characteristics** | **Concept(s) Explored** | **Perspective/ involvement explored** | **Aim(s)** | **Intervention format** | **Defined “sick day” guidance** | **Summary of Findings** | **Study Limitations** |
| --- | --- | --- | --- | --- | --- | --- | --- | --- | --- |
| Vicary et al, 2020, New Zealand, ^1^ | Quant | Total participants = 130  Participants were included if they were taking long-term medications | Current practice | Patients | To determine if a population of patients being prescribed “at risk” medications (ACEi, ARB, diuretic, NSAID and/or metformin) recalled receiving “sick day” guidance and if so, would they follow the advice. | Not applicable | **No specific advice defined.** | - Only 16 of 108 participants taking an “at risk” medication indicated that they had been advised by an HCP to stop taking medicines when they have excessive vomiting and/or diarrhoea. - Whilst half (n=8) of participants who recalled receiving advice indicated they would stop medicines if they were unwell, patients did not appear to correctly identify when they should discontinue medications. Only 3 participants would stop medications “if they have excessive vomiting and/or diarrhoea”. - Participants who recalled receiving advice may not correctly discontinue all “at risk” medications. - Many participants will seek additional guidance if they have severe vomiting and/or diarrhoea. 36% of patients (n=47) would contact the doctor or nurse and 26% (n=34) would contact their pharmacist. | - Large numbers of participants were recruited from an area that likely had increased awareness of the effects of acute illness due to a bacterial outbreak occurring in the water supply of the area. - Unable to distinguish between if no advice was provided or if patients could not recall. |
| Vicary et al, 2020, New Zealand, ^2^ | MM | Total participants = 113  Patients interviewed for follow up = 93  Participants were included if they were over 17 years of age and receiving a regular prescription for an ACEi/ARB, a diuretic, a NSAID and/or metformin. | Development, usability, or implementation of sick day management | Patients | To explore the implementation of pharmacist-led intervention aimed at educating patients on self-management during acute dehydrating illness. | Patient handout & dialogue from HCP  Pharmacist provided **verbal** AKI information and advice on self-management during acute dehydrating illness, including medicine withholding. Patients were also provided with take-home information. | If patients become **s**ick or unable to drink enough fluid to keep hydrated, (excessive vomiting (the spews), diarrhoea (the runs) or if they are experiencing fevers, sweats, and shaking), they should stop certain medications until 48 hrs of no illness. Patients should stop blood pressure tablets, water tablets, diabetes tablets, pain killers, NSAIDS, and arthritis medicines. Patients should seek medical attention if they are unwell for over 24 hours. If you are not better after 24 hours, it is very important that you seek medical attention. | - 58% (n=54) of participants interviewed on follow up recall receiving the intervention and 55% (n=42) had retained the guidance sheet provided. - Very few participants recalled the importance of maintaining hydration during acute illness. - Most patients felt confident that they could determine when they were sufficiently unwell to activate the protocol. - Around half of patients would discontinue medications during acute illness but may not cease the correct medications. - 67% (n=39) of patients were comfortable that they knew when to restart their medications but only 15 would restart medications after being symptom-free for 48 hrs as directed in the guidance. - Many patients indicated that they would contact the GP sooner than advised (after 24 hrs of no improvement or worsening symptoms), many would contact their GP within the first 24 hrs of acute illness. - Pharmacists were effective educators and well placed in the community to give sick day management advice | - Study reported on intended actions and may not reflect actual practice |
| Vicary et al., 2020, New Zealand, ^3^ | MM | Total participants= 69  Pharmacists=32  GPs=37 | Current practice | GPs  Pharmacists | To determine current practices and HCP’s perspectives around providing patient education on discontinuing “at risk” medications during acute dehydrating illness. | Not applicable | **No specific advice defined.** | - Most GPs and community pharmacists reported they had expertise to provide “sick day” patient education. - Neither pharmacists nor doctors regularly provide “sick day” advice to patients prescribed an ACEi/ARB/NSAID/diuretic - Barriers included time constraints, unclear renumeration and lack of existing GP and pharmacist collaboration regarding “sick day” guidance. - The role of all HCPs involved with patients needs to be clear, and a unified message should be agreed upon - There are different and unclear expectations of the level of patient education that should be provided by pharmacists – over half of GPs expected “sick day” guidance to be provided by pharmacists but this does not occur routinely. | - Study did not include the perspective of practice nurses. - Responses reflect perceptions rather than actual actions. |
| Morris et al., 2016, United Kingdom, ^4^ | Qual | Total participants = 42  GPs=12  Practice Nurses = 8  Pharmacists =12  Patients =10  Patients were included if they had diagnosed stage 3 CKD and been prescribed an  ACEi or ARB | Development, usability, or implementation of sick day management | Patients  Practice Nurses  GPs  Pharmacists | To determine factors that influence the implementation of routine “sick day” guidance into primary care | Patient handout & dialogue from HCP  Practice nurses that have prior sick day interventions utilised various tools including patient leaflet and reminder cards. Practice nurses that had no prior interventions were provided a sample patient hand out. | During acute illness (flu, gastroenteritis, urinary tract infection or chest infection), if a patient has a fever (temperature of 37.5^o^C) or diarrhoea or vomiting, then discontinue medications including ACEIs, ARBs, NSAIDs, diuretics, metformin. Resume medications when patient is eating/drinking normally and/or feeling better. The guidance does not apply to minor sickness. | - Patients were uncertain if they would be able to “distinguish between the symptoms of different conditions which may affect their recognition of when to begin temporary cessation.” - Patient handouts alone were seen as insufficient and unlikely to improve outcomes. More resources are required to support sick day management interventions and the prevention of AKI. - There is a need for clarity of roles and responsibility within and across different health care professions. - Both patients and HCPs highlighted the need for a consistent message about sick day rules. - Legal and professional boundaries limited the willingness of nurses and pharmacists to implement sick day management into their practice. | - No data were obtained regarding the changes to rates of community-acquired AKI in practices that had already implemented a sick day intervention. |
| Bowman et al., 2020, United States, ^5^ | MM | Total patients = 12  Patients diagnosed with CKD were included | Development, usability, or implementation of sick day management | Patients | To develop and test the usability of a mobile tablet-based educational tool designed for low health literacy CKD patients | Digital education tool  Education through auditory explanations and clinical vignettes that show photographs of medications that should be temporarily discontinued during acute illness. Patients were provided with hypothetical scenarios to help with understanding | Certain medications (diuretics, ACEIs) should be withheld during times of volume depletion (diarrhoea, vomiting, fever) because they can be harmful to the kidneys. | - Most patients found the digital tool easy to use, helpful and would recommend it to others. - Participants found patient story examples and guiding audio to be important in helping them understand the information - Most participants were able to complete the assessment after education without critical errors. - Errors were more common in older participants (age ≥65 years old) and/or those who were less familiar with digital technology –most sill completed tasks without difficulty. - Digital education tools may be effective in educating CKD patients with low health literacy and/or older age. | - Small study population. - No follow up period - retention of information in participants unknown. |
| Martindale et al. 2017, United Kingdom, ^6^ | Qual | Total participants = 29  GPs=7  Practice nurses=5  Community Pharmacists= 5  Practice Pharmacists=4  Patients=5  Medical practice manager=1  Community pharmacist manager=1  Health-care assistant =1 | Development, usability, or implementation of sick day management | Patients  GPs  Practice Nurses  Pharmacists | To examine the implementation of sick day guidance cards designed to prevent acute kidney injury , in primary care settings. | Patient handout & dialogue from HCPs  Provision of sick day guidance cards to patients who were taking at-risk medications. | Patients should stop taking their ACEI, ARB, NSAID, diuretic or metformin when they are unwell (vomiting, diarrhoea unless minor). Patients should restart their medications after 24-48 hours of eating and drinking normally | - HCPs believed the card required sufficient health literacy to be able to assess the severity of their symptoms and when sick day management guidance should be applied. - It appears that communication between patients and HCPs must accompany patient handouts to ensure patient understanding, but accounts indicate that this did not always occur. - HCPs concerned about the limited data on outcomes or best practice is available to determine when the most appropriate time is to stop, restart and dosages to reinitiate at. - Implementing sick day management may be difficult in patients who have cognitive impairments, reduced literacy in English, visual impairments, or elderly housebound patients. - As a stand-alone intervention, sick day management in the form of patient handouts may be of limited benefit. | - Limited recounts of patient experiences despite extensive recruitment efforts. - Number of patients who received intervention is unknown. |
| Faber et al., 2019, Netherlands, ^7^ | Quant | Total participants= 562  Patients who contacted a GP reporting acute illness and were at risk of adverse events due to risk factors and/or medication use | Current Practice  Outcomes | GPs | To report on rates of discontinuation advice being offered by GPs and the incidence of complications following acute illness. | Not applicable | Patients should be advised to discontinue or adjust dosages of at-risk medications during acute illness (chills, fever, diarrhoea, gastrointestinal infection, and gastroenteritis). | - From a total of 816 acute illness episodes identified from 562 at-risk patients, GPs did not offer patients advice to discontinue or adjust medication dosages nor referred them to hospital in 91% of acute illness episodes - In 3.1% (n=25) of episodes of acute illness, a complication occurred in the subsequent 3 months after contacting their GP, most commonly AKI. - In 3 episodes, complications occured even though sick day management advice was given; 2 cases of reported AKI, and 1 case of reported hypotension. | - No statistical comparison of outcome measures between at-risk patients who continued their medication versus patients who discontinued their medication during acute illness – insufficient sample size in discontinuation group - Rationale for not providing discontinuation advice was not determined – uncertain if it was clinically appropriate to do so. |
| Doerfler et al., 2019, United States, ^8^ | MM | Total participants=20  Patients were included if they had diagnosed stage 3-5 CKD | Development, usability, or implementation of sick day management | Patients | To evaluate the use of patient handouts in patients with CKD | Patient Handout  A sick day protocol card | Patients should withhold ACEi, ARB, diuretic, NSAID and/or metformin when they have a gastrointestinal or fever-related and volume-depleting illness. Patients should restart medications after 24–48 hours of eating and drinking normally. | - Most participants could correctly identify an index scenario where sick day management advice should be applied - Around half of the time, participants incorrectly applied sick day management advice in other scenarios where it would be inappropriate to do so. - 95% made errors when selecting appropriate medications to discontinue - “Utility of sick day protocols expressed on portable index cards were disappointing and raised safety concerns around incorrect activation leading to harm.” | - Participants were tested directly after education on sick day management – this may not reflect the true performance of participants in their daily lives. |
| Whiting et al., 2017, United Kingdom, ^9^ | Systematic  Review | Total participants= 1663  Patients from across 3 RCTs and 3 prospective cohort studies | Outcomes | Not applicable | To summarise evidence on discontinuing at-risk medications to prevent AKI. | Not applicable | **No specific advice defined.** | - No published studies evaluated the impact of temporarily discontinuing medications during acute illness on the incidence of AKI. - There was no indication of ongoing studies exploring the outcome of sick day management interventions. | - Conclusions of the systematic review are limited by quality and quantity of studies available on topic |
| Fink et al., 2022, United States | RCT | Total participants = 315  Participants receiving intervention = 159  Patients were included if they had stage 3-5 CKD and were currently taking a renin-angiotensin-aldosterone inhibitor, NSAID or metformin | Outcomes | Patients | To evaluate the impact of a sick day protocol on clinical outcomes of patients with existing CKD | Patient Handout & Digital tool  For all patients in the intervention arm, a sick day protocol card was provided, outlining temporary discontinuation advice. An interactive voice response system contacted patients weekly and in the instance that a sick day event was reported, relevant advice was provided over the telephone. | Patients should stop taking their ACEis, ARBs, diuretics, metformin or NSAIDs if they are unwell with vomiting, diarrhoea, fever, sweats and/or shaking. They should restart 24-48 hours after eating and drinking normally. If they are in doubt, contact a pharmacist, doctor, or nurse. | - When adjusted for baseline eGFR, there was no statistical difference in the mean change in eGFR from baseline to 6 months between patients in the sick day protocol and usual care group - There was no significant increase in the adjusted prevalence ratio of hospitalisations in the sick day protocol and usual care group (prevalence ratio, 1.30; 95% CI, 0.96-1.76) - There was a statistical difference in number of hospitalisations, emergency department, or urgent care visits between the sick day protocol and usual care group - 50% (n=33) of sick-day events reported through the interactive voice response system were true sick days. The other 50% consisted of erroneous data (n=19) or misclassified medical events (n=14) - Of the patients that experienced a true sick day event in the sick day protocol group, 49.2% (n=14) correctly discontinued their medication | - There was low incidence of reported sick days and AKI events across both arms of the RCT. - No blood tests were scheduled throughout the 6-month period – instances of subclinical AKI may not have been reported or detected |

**Abbreviations:** ACEI – angiotensin converting enzyme inhibitor, ARB – angiotensin receptor blocker, AKI – acute kidney injury, CKD – chronic kidney disease, HCP – health care professional, MM – mixed method design, NSAID – non-steroidal anti-inflammatory drug, Quant – quantitative design, Qual – qualitive design, RCT – randomised controlled trial

**APPENDIX 4:** Characteristics of guidelines describing sick day management

| **Guideline, Organisation, Reference** | **Year, Country** | **Recommendation(s)** | **Evidence and/or Rationale** | **Strength of Recommendation** |
| --- | --- | --- | --- | --- |
| The assessment & management of chronic kidney disease in adults,  National Clinical Guidelines for Qatar, ^10^ | Qatar, 2020 | - “Advise anyone with CKD about the increased risk of AKI if they become acutely ill” - “Temporarily discontinue potentially nephrotoxic and renally excreted drugs in patients with an eGFR of <60mL/min/1.73m^2^ who have a serious intercurrent illness that increases the risk of AKI” | - Expert Opinion - Reference to **“**KDIGO 2012 Clinical Practice Guideline for the Evaluation and Management of Chronic Kidney Disease”, see ^11^ | - Moderate certainty of a net benefit from the recommendation. |
| Acute kidney injury: prevention, detection, and management.  National Institute for Health and Care Excellence (NICE), ^12^ | UK, 2019 | - “Consider temporarily stopping ACE inhibitors and ARBs in adults, children, and young people with diarrhoea, vomiting or sepsis until their clinical condition has improved and stabilised.” | - Reference to 2013 iteration of guideline, see  **“**Acute kidney injury: Prevention, detection and management up to the point of renal replacement therapy”, see ^13^ | - Not provided |
| Acute kidney injury: Prevention, detection and management up to the point of renal replacement therapy  National Institute for Health and Care Excellence (NICE), ^13^ | UK, 2013 | - “Patients should be advised to cease their ACEI or ARB, with any hypovolaemic illness (e.g. diarrhoea and/or vomiting, hypotension) or major infection. The suspension of ACEI or ARB therapy should last until they are clearly improving.” | - Expert consensus - **Rationale:** No comparative evidence was identified for continuing versus stopping ACEIs and/or ARBs during acute illness but continual use of ACEIs/ARBs during sepsis or diarrhoea and vomiting is clearly associated with AKI. Temporary discontinuation of ACEIs/ARBs is unlikely to greatly increase the risk of cardiovascular events | - Not provided |
| KDIGO 2012 Clinical Practice Guideline for the Evaluation and Management of Chronic Kidney Disease, ^11^ | International, 2013 | - “Recommend temporary discontinuation of potentially nephrotoxic and renally excreted drugs in people with a GFR < 60 ml/min/1.73 m^2^ who have serious intercurrent illness that increases the risk of AKI. These agents include but are not limited to: RAAS blockers (including ACEI’s, ARBs, aldosterone inhibitors, direct renin inhibitors), diuretics, NSAIDs, metformin, lithium, and digoxin.” | - **Rationale**: Toxicity of certain medications may worsen in acute illness, particularly if there is a risk of dehydration such as diarrhoea and vomiting. - Reference to evidence not explicit | - “Low quality of evidence available and effect may be substantially different from the estimate of the effect, but most patients should receive the recommended course of action.” |
| Chronic Kidney Disease in Diabetes  Canadian Diabetes Association, ^14^ | Canada, 2018 | - “Adults with diabetes and CKD should be given a “sick day” medication list that outlines which medications should be held during times of acute illness: | - Expert Consensus - **Rationale:** Use of certain medications during acute illness associated with dehydration from reduced oral intake or vomiting and/or diarrhoea may lead to kidney injury | - Not provided. |
| UK Kidney Association Clinical Practice Guideline: Sodium-Glucose Co-transporter-2 (SGLT-2) Inhibition in Adults with Kidney Disease  The UK Kidney Association, ^15^ | United Kingdom, 2021 | - Patients to receive sick day management advice when initiated on SGLT-2 inhibitors and should be reminded of sick day management at every medication review - Exemplar patient information sheet provided - “If you are unwell (vomiting, diarrhoea, fever, sweats and shaking), you should temporarily miss out on … blood pressure pills, diuretics, SGLT-2 inhibitor. Restart medicines as soon as you are well and eating normally. Seek medical advice if you continue to feel unwell after 48 hours.” | - **Rationale:** SGLT-2 inhibitors have a “combined osmotic diuretic and natriuretic effect” that may increase the risk hypovolaemia and AKI - Discussion of low-quality evidence by reference to evidence not explicit | - Strong recommendation to implement advice as benefits clearly outweigh the risks for most, if not all patients |
| Chronic Kidney Disease (CKD) Management in Primary Care  Kidney Health Australia, ^16^ | Australia, 2020 | - Patients should withhold ACEI, ARBs, NSAIDs, diuretics, SGLT-2 inhibitors, metformin, sulfonylureas if they become acutely ill and are unable to maintain adequate fluid intake - Medications should be re-initiated when the patient’s clinical condition stabilises | - Reference to evidence not explicit | - Not provided. |

**Abbreviations:** ACEI – angiotensin converting enzyme inhibitor, ARB – angiotensin receptor blocker, AKI – acute kidney injury, CKD – chronic kidney disease, NSAID – non-steroidal anti-inflammatory drug

**References**

1. Vicary D, Hutchison C, Aspden T. Preventing acute kidney injury: assessing awareness to temporarily discontinue 'at-risk' medicines during acute illness in a New Zealand cohort. *N Z Med J*. 07 31 2020;133(1519):12-23.

2. Vicary D, Hutchison C, Aspden T. Demonstrating the value of community pharmacists in New Zealand educating a targeted group of people to temporarily discontinue medicines when they are unwell to reduce the risk of acute kidney injury. *International Journal of Pharmacy Practice*. Dec 2020;28(6):569-578. doi:<https://dx.doi.org/10.1111/ijpp.12666>

3. Vicary D, Hutchison C, Aspden T. Avoiding acute kidney injury in primary care: attitudes and behaviours of general practitioners and community pharmacists in Hawke’s Bay. *Journal of Primary Health Care*. 2020;12(3):244. doi:10.1071/hc19106

4. Morris RL, Ashcroft D, Phipps D, et al. Preventing Acute Kidney Injury: a qualitative study exploring 'sick day rules' implementation in primary care. Research Support, Non-U.S. Gov't. *BMC Fam Pract*. 07 22 2016;17:91. doi:<https://dx.doi.org/10.1186/s12875-016-0480-5>

5. Bowman C, Lunyera J, Alkon A, et al. A Patient Safety Educational Tool for Patients With Chronic Kidney Disease: Development and Usability Study. *JMIR Form Res*. May 28 2020;4(5):e16137. doi:<https://dx.doi.org/10.2196/16137>

6. Martindale AM, Elvey R, Howard SJ, McCorkindale S, Sinha S, Blakeman T. Understanding the implementation of 'sick day guidance' to prevent acute kidney injury across a primary care setting in England: a qualitative evaluation. *BMJ Open*. Nov 08 2017;7(11):e017241. doi:<https://dx.doi.org/10.1136/bmjopen-2017-017241>

7. Faber SJ, Scherpbier ND, Peters HJG, Uijen AA. Preventing acute kidney injury in high-risk patients by temporarily discontinuing medication - an observational study in general practice. Observational Study. *BMC Nephrology*. 12 04 2019;20(1):449. doi:<https://dx.doi.org/10.1186/s12882-019-1636-z>

8. Doerfler RM, Diamantidis CJ, Wagner LA, et al. Usability Testing of a Sick-Day Protocol in CKD. *Clinical Journal of The American Society of Nephrology: CJASN*. 04 05 2019;14(4):583-585. doi:<https://dx.doi.org/10.2215/CJN.13221118>

9. Whiting P, Morden A, Tomlinson LA, et al. What are the risks and benefits of temporarily discontinuing medications to prevent acute kidney injury? A systematic review and meta-analysis. *BMJ Open*. 04 07 2017;7(4):e012674. doi:<https://dx.doi.org/10.1136/bmjopen-2016-012674>

10. National Clinical Guideline: The Assessment and Management of Chronic Kidney Disease in Adults (2020).

11. Levin A, Stevens PE, Bilous RW, et al. Kidney Disease: Improving Global Outcomes (KDIGO) CKD Work Group. KDIGO 2012 clinical practice guideline for the evaluation and management of chronic kidney disease. *Kidney international supplements*. 2013;3(1):1-150.

12. UK GUT. Acute kidney injury: prevention, detection and management. 2019;

13. National Clinical Guideline C. National Institute for Health and Clinical Excellence: Guidance. *Acute Kidney Injury: Prevention, Detection and Management Up to the Point of Renal Replacement Therapy*. Royal College of Physicians (UK)

Copyright © 2013, National Clinical Guideline Centre.; 2013.

14. Philip McFarlane DC, Richard E. Gilbert, Peter Senior. Diabetes Canada 2018 Clinical Practice Guidelines for the Prevention and Management of Diabetes in Canada: Chronic Kidney Disease in Diabetes

2018;42(1)

15. Association TUK. *UK Kidney Association Clinical Practice Guideline: Sodium-Glucose Co-transporter-2 (SGLT-2) Inhibition in Adults with Kidney Disease*. 2021.

16. Australia KH. *Chronic Kidney Disease (CKD) Management in Primary Care*. 2020. <https://kidney.org.au/uploads/resources/CKD-Management-in-Primary-Care_handbook_2020.1.pdf>
